# Inflammatory turmoil within: an exploration of autoinflammatory disease genetic underpinnings, clinical presentations, and therapeutic approaches

**DOI:** 10.1186/s42358-024-00404-9

**Published:** 2024-08-22

**Authors:** Kátia Tomie Kozu, Renan Rodrigues Neves Ribeiro do Nascimento, Patrícia Pontes Aires, Rafael Alves Cordeiro, Thais Costa Lima de Moura, Flavio Roberto Sztajnbok, Ivanio Alves Pereira, Adriana Almeida de Jesus, Sandro Félix Perazzio

**Affiliations:** 1 Universidade de Sao Paulo, Faculdade de Medicina (USP FM), Sao Paulo, Brazil; 2 Universidade Federal de Sao Paulo, Escola Paulista de Medicina (Unifesp EPM), Rua Otonis, 863, Vila Clementino, São Paulo, SP 04025-002 Brazil; 3 Federal University of Rio de Janeiro: Universidade Federal do Rio de Janeiro, Rio de Janeiro, Brazil; 4 Universidade do Sul de Santa Catarina (Unisul), Florianópolis, Brazil; 5 National Institutes of Health (NIH), Bethesda, USA; 6 Division of Immunology and Rheumatology, Fleury Laboratories, Sao Paulo, SP Brazil

**Keywords:** Monogenic, Autoinflammatory diseases, Inflammasomopathies, Relopathies, IL-18/IL-36 signaling pathway defects, Type I interferonopathies, Anti-inflammatory signaling pathway impairment

## Abstract

Systemic autoinflammatory diseases (SAIDs) arise from dysregulated innate immune system activity, which leads to systemic inflammation. These disorders, encompassing a diverse array of genetic defects classified as inborn errors of immunity, are significant diagnostic challenges due to their genetic heterogeneity and varied clinical presentations. Although recent advances in genetic sequencing have facilitated pathogenic gene discovery, approximately 40% of SAIDs patients lack molecular diagnoses. SAIDs have distinct clinical phenotypes, and targeted therapeutic approaches are needed. This review aims to underscore the complexity and clinical significance of SAIDs, focusing on prototypical disorders grouped according to their pathophysiology as follows: (i) inflammasomopathies, characterized by excessive activation of inflammasomes, which induces notable IL-1β release; (ii) relopathies, which are monogenic disorders characterized by dysregulation within the NF-κB signaling pathway; (iii) IL-18/IL-36 signaling pathway defect-induced SAIDs, autoinflammatory conditions defined by a dysregulated balance of IL-18/IL-36 cytokine signaling, leading to uncontrolled inflammation and tissue damage, mainly in the skin; (iv) type I interferonopathies, a diverse group of disorders characterized by uncontrolled production of type I interferons (IFNs), notably interferon α, β, and ε; (v) anti-inflammatory signaling pathway impairment-induced SAIDs, a spectrum of conditions characterized by IL-10 and TGFβ anti-inflammatory pathway disruption; and (vi) miscellaneous and polygenic SAIDs. The latter group includes VEXAS syndrome, chronic recurrent multifocal osteomyelitis/chronic nonbacterial osteomyelitis, Schnitzler syndrome, and Still’s disease, among others, illustrating the heterogeneity of SAIDs and the difficulty in creating a comprehensive classification. Therapeutic strategies involving targeted agents, such as JAK inhibitors, IL-1 blockers, and TNF inhibitors, are tailored to the specific disease phenotypes.

## Introduction

Systemic autoinflammatory diseases (SAIDs) are clinical conditions caused by defects that induce uncontrolled hyperactivation and dysregulation mainly within the innate immune system. These disorders are characterized by persistent or recurrent systemic inflammation, elevated acute-phase reactants without discernible causes, and indicators of autoimmunity, such as self-reactive T cells or the production of autoantibodies [1]. Although previously associated to fever episodes within specific periodic intervals, which defined some syndromes, current understanding broadens this concept to conditions with unremarkable febrile or even unfebrile patterns. Therefore, specific diagnostic protocols focused on fever periodicity are strongly discouraged. SAIDs fall under a diverse category of genetic disorders known as inborn errors of immunity (IEI). The IEI category currently encompasses a spectrum of 485 distinct monogenic diseases listed in the latest classification of the International Union of Immunological Societies [2]. Although conventionally associated with susceptibility to recurrent infections, recent understanding has extended the phenotypic spectrum of IEI to include autoimmune diseases, severe allergic phenomena, lymphoproliferative disorders, and SAIDs. In these scenarios, there is primarily a disruption in immune system regulation, prompting the recent introduction of the term primary immunoregulatory diseases to distinguish them, constituting approximately 30% of IEI [3].

Advances in genetic sequencing accessibility and the efficient application of these technologies are propelling the exponential increase in gene discovery of new clinically relevant variants. Nevertheless, certain limitations persist in identifying cases, as demonstrated by the existence of diverse genetic variants within the same gene, resulting in significant variations in disease severity and clinical manifestations. The complexity of identifying and understanding SAIDs arises from a multitude of genetic factors, which are not necessarily mutually exclusive. These factors include the Mendelian inheritance pattern (dominant, recessive, haploinsufficiency, or dominant-negative), the variant penetrance, the embryonic origin of the variant (germline or somatic), the predominantly affected cell cluster, the presence of mosaicism, and the functional characteristics (gain or loss of function and total or partial) [4]. Furthermore, despite the characterization of more than 80 SAIDs, approximately 40% of patients with evident autoinflammatory clinical phenotypes are without a defined molecular diagnosis. The rarity of these conditions understandably complicates their recognition and the development of classification criteria. Moreover, the identification of variants of unknown significance may not determine a definite molecular diagnosis, especially when associated with an atypical clinical phenotype. However, early diagnosis proves pivotal in averting long-term complications stemming from recurrent inflammation. The striking heterogeneity and fast-growing number of SAIDs make it difficult to gather information on all SAIDs within a single review, which was not our pretension.

The main purpose of our review is to call the attention of rheumatologists to this important group of disorders and invite them to delve into the more specialized literature. Moreover, a comprehensive classification encompassing all SAIDs is challenging; therefore, this review is focused on prototypical disorders divided according to their pathophysiological similarities as follows:

- Inflammasomopathies;
- Relopathies;
- IL-18/IL-36 signaling pathway defect-induced SAIDs;
- Type I interferonopathies;
- Anti-inflammatory signaling pathway impairment-induced SAIDs;
- Miscellaneous and polygenic SAIDs.

## Inflammasomopathies

**Fig. 1:**
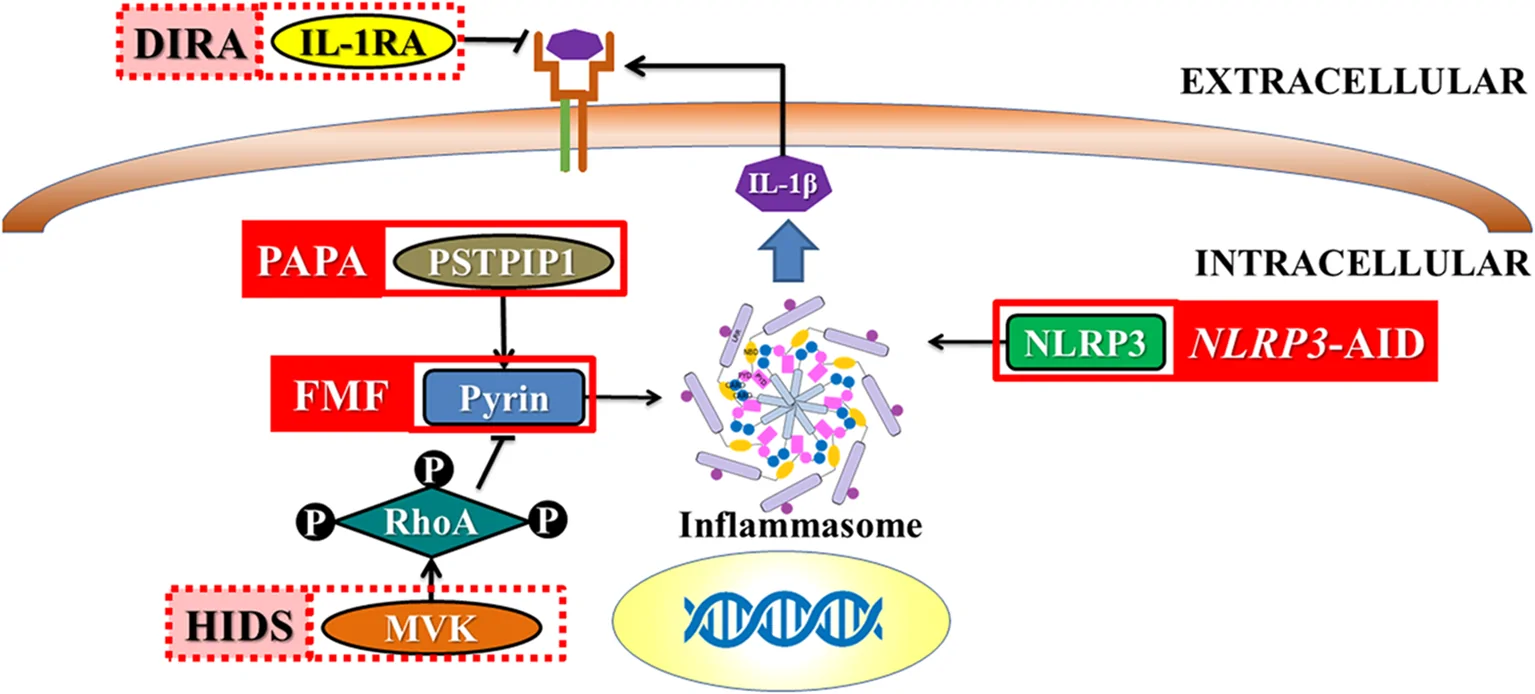
Main inflammasomopathies classified according to pathophysiology. Inflammasome assembly may be triggered by different pathways but mostly culminates with the activation of IL-1β, which plays autocrine and paracrine roles in cell activation. PSTPIP1 activates pyrin inflammasome assembly. By directly modulating pyrin activation, MVK-phosphorylated Rhoa is a pivotal regulator of this pathway. NLRP3 is the main component of a distinct inflammasome. Red squares depict the main syndromes and their respective molecular targets classified as gain-of-function (continuous line) and loss-of-function (dashed line)-causing mutations. DIRA (deficiency of IL-1β receptor antagonist); IL-RA (IL-1β receptor antagonist); FMF (familial Mediterranean fever); HIDS (hyper-IgD syndrome); MVK (mevalonate kinase); NLRP3-AID (nucleotide-binding domain, leucine-rich repeat family, pyrin domain containing 3-associated autoinflammatory disease); PAPA (pyogenic arthritis, pioderma gangrenosum and acne syndrome); PSTPIP1 (proline-serine-threonine phosphatase interacting protein 1); RhoA (Ras homolog family member A)

Inflammasomopathies (a.k.a., IL-1β-activation syndromes) are individually the most frequent SAIDs and represent most of the classic periodic fever syndromes characterized by excessive activation of inflammasomes, which induce notable IL-1β release [5]. The release of IL-1β requires two steps: (i) pro-IL-1β transcription, which is usually induced by alarmins or pathogen-associated molecular patterns; and (ii) inflammasome assembly and cleavage of pro-IL-1β into active IL-1β. Inflammasomes, which are composed of sensor and effector molecules, play a pivotal role in orchestrating inflammatory responses. Figure 1 summarizes the main inflammasomopathies according to pathophysiology, and Table 1 provides an extended list of IL-1β-activation syndromes.

**Table 1:** Main monogenic inflammasomopathies/IL-1β-activation syndromes

Disorder	Gene	Inheritance	OMIM	Clinical features
FMF/PAAD	*MEFV*	AR/AD	249100/134610	Fever, serositis, pleural effusion peritonitis, arthritis, erysipelas-like erythema (rash)
HIDS/MKD	*MVK*	AR	260920/610377	Fever, abdominal pain, lymphadenopathy, rash, oral ulcers
PAPA syndrome	*PSTPIP1*	AD	604416	Pyoderma gangrenosum, aseptic pyogenic arthritis, rash, neutropenia, hepatosplenomegaly
NLRP3-AID	*NLRP3*	AD GOF	120100 (mild)	Cold urticaria, arthralgia, fever, conjunctivitis
			191900 (moderate)	Urticarial rash, arthritis, deafness, conjunctivitis, fever
			607115 (severe)	Urticarial rash, aseptic meningitis, deafness, optical nerve atrophy, fever, epiphyseal growth
DIRA	*IL-1RN*	AR	612852	Pustular rash, osteomyelitis, periostitis and fever
PLAID	*PLCG2*	AD GOF	614878/614468	Cold urticaria hypogammaglobulinemia, impaired humoral immunity, autoinflammation
NLRP4-AID	*NLRP4*	AR	NA	Cold urticaria, arthralgia, fever, CNS involvement
NLRP1 deficiency	*NLRP1*	AR	617388	Dyskeratosis, autoimmunity and arthritis
NLRP1-GOF	*NLRP1*	AD GOF	615225	Palmoplantar carcinoma, corneal ulcer, arthritis, recurrent respiratory papillomatosis

AD (autosomal dominant); AR (autosomal recessive); CNS (central nervous system); DIRA (deficiency of IL-1 receptor antagonist); HIDS (hyper-IgD syndrome); FMF (familial Mediterranean fever); GOF (gain-of-function); MKD (mevalonate kinase deficiency); NA (not available); NLRP3-AID (nucleotide-binding domain, leucine-rich repeat family, pyrin domain containing 3-associated autoinflammatory diseases); NLRP4-AID (nucleotide-binding domain, leucine-rich repeat family, pyrin domain containing 4-associated autoinflammatory diseases); PAAD (pyrin-associated autoinflammatory disease); PAPA (pyogenic arthritis, pyoderma gangrenosum and acne); PLAID (1-phosphatidylinositol-4,5-bisphosphate phosphodiesterase gamma-2-associated antibody deficiency and immune dysregulation)

### Pyrin-associated autoinflammatory diseases (PAAD)

Familial Mediterranean fever (FMF) is the most prevalent hereditary SAIDs and typically manifests as an autosomal recessive condition stemming from gain-of-function (GOF) mutations within the *MEFV (*mediterranean fever) gene. More recently, patients with FMF manifestations and autosomal dominant inheritance were identified, broadening the spectrum of the disease to PAAD [6]. *MEFV* encodes pyrin, a critical protein that is instrumental in assembling the pyrin inflammasome and is pivotal for modulating IL-1β production and interactions with caspase-1 and other inflammasome constituents.

FMF/PAAD mainly affects descendants from the Eastern Mediterranean and along the ancient “silk route”, encompassing Sephardic Jews, Armenians, and individuals of Turkish and Arab descent [7, 8]. Over 300 mutations in the *MEFV* gene have been identified, with an estimated 10% deemed pathogenic [9]. The onset of FMF frequently manifests during childhood, with episodic flares typically lasting one to three days and recurring intermittently, varying from weekly to once every few years, interspersed with symptom-free intervals. Clinical presentations include peritonitis, abdominal symptoms mimicking a surgical acute abdomen, nonerosive oligoarthritis, pleuritis, pericarditis, scrotal pain, and aseptic meningitis. Skin involvement is found in 12–40% of patients, often manifest as erysipelas-like rashes on the lower extremities. Triggers for flares may include stress, exercise, infections, and even pregnancy [9, 10].

Laboratory investigations during flares often reveal elevated acute-phase reactants, such as the erythrocyte sedimentation rate (ESR), C-reactive protein (CRP), and serum amyloid A (SAA), as well as leukocytosis. Recurrent episodes of systemic inflammation predispose FMF patients to secondary or reactive A amyloidosis (AA). This occurs because the liver produces excessive insoluble serum amyloid A (SAA) protein upon stimulation by proinflammatory cytokines. Colchicine represents a mainstay in managing and preventing flares and amyloidosis development. Anti-IL-1 agents, such as anakinra, rilonacept and canakinumab, which is FDA-approved for FMF, are often used for colchicine-refractory cases [11, 12]. Interestingly, anti-IL-6 had also reduced the frequency of inflammatory episodes in patients with FMF and AA [13]. Based on expert opinion, colchicine resistance is defined as persistent disease activity represented by one or more flares per month over a 3-month period and/or elevated inflammatory markers between attacks in the absence of alternative explanations [14].

### Cryopyrin-associated periodic syndrome (CAPS)

CAPS [a.k.a. *NLRP3* (nucleotide-binding domain, leucine-rich repeat family, pyrin domain containing 3)-associated autoinflammatory diseases (NLRP3-AID)] represent a cluster of spectral autosomal dominant disorders characterized by *NLRP3* GOF mutations [1, 15], including familial cold autoinflammatory syndrome (FCAS), Muckle-Wells syndrome (MWS), and NOMID/CINCA (neonatal-onset multisystem inflammatory disease/chronic infantile neurological cutaneous and articular) syndrome. The updated taxonomy respectively classifies these conditions as mild, moderate, or severe *NLRP3*-AID [1]. *NLRP3* is a pivotal cryopyrin inflammasome component.

Mild *NLRP3*-AID/FCAS typically manifests as short-lived (1–2 days) episodes of fever following exposure to cold temperatures (1–2 h postexposure). Patients may present other intermittent symptoms, such as rash, myalgia, fatigue, and headaches. Amyloidosis remains rare.

Patients with moderate *NLRP3*-AID/MWS present with periodic fever episodes (1–3 days), urticarial rash, myalgia, progressive sensorineural hearing loss, and a greater predisposition to amyloidosis. Additionally, ocular manifestations such as uveitis, conjunctivitis, and episcleritis can occur.

Severe *NLRP3*-AID/NOMID manifests as a classic triad of urticarial rash, chronic aseptic meningitis, and arthropathy derived from abnormal epiphyseal calcification and cartilage overgrowth, leading to joint deformities. Premature patellar ossification and overgrowth are characteristics of *NLRP3*-AID. Chronic cochlear inflammation precipitates Corti cell atrophy, contributing to hearing loss. Chronic aseptic meningitis can lead to increased intracranial pressure, hydrocephalus, papilledema, brain atrophy, severe cognitive impairment, optic nerve atrophy, and potential vision loss [16].

Despite the clinical diversity among *NLRP3*-AID patients, IL-1 blockade consistently yields dramatic and consistent responses. Higher doses of IL-1 blockers may be necessary for effective management of severe cases [16]. Anakinra (NOMID), canakinumab (FCAS and MWS) and rilonacept (FCAS and MWS) are all currently FDA-approved for the treatment of CAPS.

### Hyperimmunoglobulinemia D and periodic fever syndrome - Hyper IgD syndrome (HIDS)/mevalonate kinase deficiency (MKD)

HIDS arises from autosomal recessive mutations in *MVK*, which encodes mevalonate kinase, a pivotal enzyme in the cholesterol synthesis pathway. A decrease in the enzymatic activity of mevalonate kinase (1–10% of normal levels) significantly impacts the metabolic pathway and contributes to the phenotypic manifestation of HIDS.

Typically, HIDS episodes manifest before the age of one year and last three to seven days, recurring at intervals of four to six weeks. Vaccinations can act as triggering events for these episodes, albeit the decision whether immunization should be analyzed individually, considering the risk for disease flares and vaccine-induced disease. Overall, similarly to other rheumatic immune-mediated diseases under immunosuppressant therapy, live vaccines should be avoided [17, 18].

Patients may also present with polyarthralgia or nonerosive arthritis affecting large joints; cervical lymphadenopathy; sore throat and oral ulcers; hepatosplenomegaly and abdominal manifestations such as pain, vomiting, and diarrhea; and variable skin lesions, including maculopapular, urticarial, nodular, and purpuric rashes. Gastrointestinal symptoms are a cardinal feature of MKD. Additionally, although uncommon, MKD may possibly impose susceptibility to severe infection, especially under immunosuppressant treatment or in association with other comorbidities [19]. In contrast, MVK activity impairment of less than 1% correlates with the severe phenotype of mevalonic aciduria, characterized by profound neurological compromise, myopathy, and an ominous prognosis [20]. Despite the historical association of high IgD levels in HIDS, serum quantification has limited diagnostic yield. The therapy of choice involves IL-1 blockade [21], such as canakinumab, which is FDA-approved for the treatment of HIDS/MKD. Statins and leukotriene inhibitor are anecdotally used in the treatment of MKD patients [21].

### Pyogenic arthritis, pyoderma gangrenosum and acne (PAPA) syndrome

PAPA syndrome is an autosomal dominant disease with variable penetrance caused by heterozygous missense variants in *PSTPIP1* (proline-serine-threonine phosphatase interacting protein 1). Mutated PSTPIP1 undergoes hyperphosphorylation, resulting in uncontrolled activation of pyrin and the inflammasome [22]. Patients may exhibit neutrophilic dermatosis and aseptic arthritis, particularly affecting the elbows, knees, and ankles. The aseptic arthritis is more common in the first two decades of life, characterized by neutrophilic infiltrate and triggered by minor traumas or spontaneously. Joint erosions and deformities can occur in long-term untreated patients. Typically, joint symptoms tend to decrease and there is a predominance of cutaneous manifestations in young adults. Pathergy, nodulocystic acne, and pyoderma gangrenosum are frequent manifestations. The clinical presentation can be diverse, with variations in the disease described based on the presence of suppurative hidradenitis or psoriatic arthritis [23]. Regardless of the phenotype, PAPA syndrome patients often exhibit increased inflammatory marker levels, especially during flares. Currently, TNF inhibitors and IL-1 blocking agents are currently the most effective treatments. However, given the emerging evidence on the role of IL-17 in PAPA pathogenesis, IL-17 antagonists may also be helpful.

### Deficiency of IL-1 receptor antagonist (DIRA)

DIRA is a rare autosomal recessive autoinflammatory disorder characterized by a deficiency in the naturally occurring extracellular IL-1 receptor antagonist (IL-1Ra). Consequently, unopposed IL-1 signaling triggers exaggerated inflammatory cascades. Clinically, DIRA manifests with early-onset recurrent episodes of systemic inflammation, usually with neonatal onset of pustular dermatosis, periostitis and non-infectious recurrent osteomyelitis, fever, arthritis, bone deformities and serositis. Patients commonly present with musculoskeletal symptoms, including joint pain and swelling. Timely and accurate diagnosis is crucial for effective management. The main treatment strategy is IL-1 blockade to mitigate exaggerated inflammatory responses. Anakinra, a recombinant IL-1Ra, and rilonacept, a soluble IL-1 receptor, have been demonstrated to be efficacious in ameliorating symptoms and preventing disease flares [24, 25].

## Relopathies

**Fig. 2:**
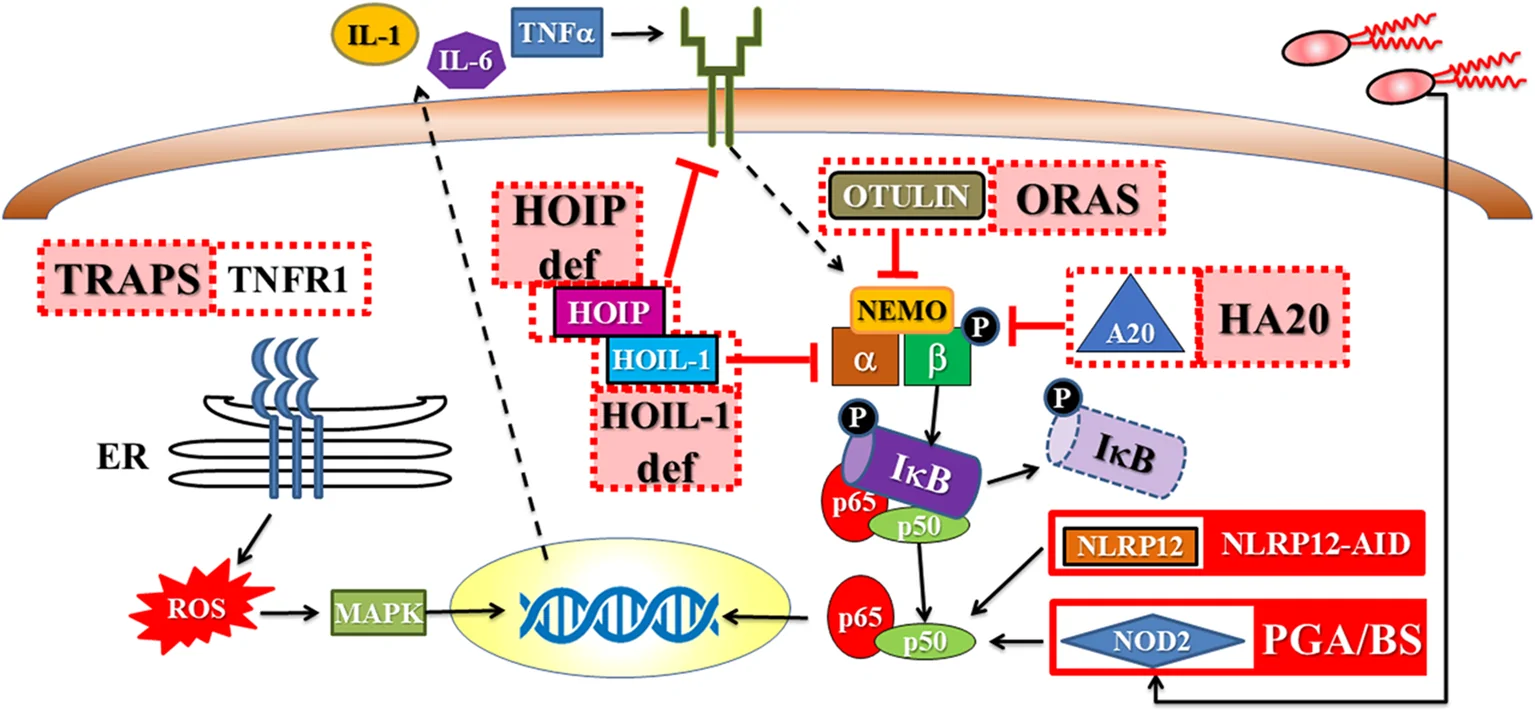
Main relopathies classified according to pathophysiology. Cytokine receptor dimerization induced by its specific ligand (e.g., TNFα) initiates canonical NF-κB pathway signaling. The activation of cytokine receptor intracellular signaling is mediated by the activation of the IKK complex, which is composed of three subunits: α, β, and g (a.k.a. NEMO). This process may be modulated by different proteins in a cascade of ubiquitination events: A20, OTULIN, HOIL-1 and HOIP. The activated IKK complex phosphorylates IκB, which signals its degradation and releases NF-κB1, a protein dimer composed of p50 and p65 (a.k.a. RelA). NF-κB1 may also be activated by intracellular pattern recognition receptors, such as NOD2, and directly by NLRP12. As a potent proinflammatory transcription factor, NF-κB1 induces cytokine (such as IL-1, IL-6 and TNFα) and proliferation-related genes. TRAPS involves intracellular ROS production induced by the accumulation of misfolded mutated TNFR1 in the ER in a process known as the unfolded protein response, culminating in MAPK activation. Red squares depict the main syndromes and their respective molecular targets classified as gain-of-function (continuous line) and loss-of-function (dashed line)-causing mutations. ER (endoplasmic reticulum); HA20 (haploinsufficiency of A20); HOIL-1 (Heme-oxidized IRP2 ubiquitin ligase 1); HOIP (HOIL-1 interacting protein); HOIL-1 def (HOIL-1 deficiency); HOIP def (HOIP deficiency); IκB (inhibitor of NF-κB); IKK (IκB kinase kinase); MAPK (mitogen-activated protein kinase); IL-1 (interleukin 1); IL-6 (interleukin 6); NEMO (NF-κB essential modulator); NF-κB1 (nuclear factor κB1); NLRP12 (NACHT, LRR and PYD domains-containing protein 2); NLRP12-AID (NLRP12-associated autoinflammatory disease); NOD2 (nucleotide-binding oligomerization domain containing 2); ORAS (OTULIN-related autoinflammatory syndrome); OTULIN (OTU deubiquitinase with linear linkage specificity); PGA/BS (pediatric granulomatous arthritis/Blau syndrome); ROS (reactive oxygen species); TNFα (tumor necrosis factor α); TNFR1 (TNFα receptor 1); TRAPS (tumor necrosis factor receptor-associated periodic syndrome)

Relopathies encompass a group of monogenic disorders characterized by dysregulation of the nuclear factor-kappa B (NF-κB) signaling pathway, resulting in sustained inflammatory responses and impaired cellular homeostasis; these disorders are also known as NF-κB activation syndromes. NF-κB is a ubiquitous crucial transcription factor involved in the regulation of multiple immune responses, apoptosis, and inflammation. The term “relopathy” is derived from the NF-κB subunits homo- or heterodimers, hence they are also identified as RelA, RelB, and c-Rel. Although IL-1β is an important effector, TNFα and IL-6 also play pivotal roles in the pathogenesis of most of these disorders. Figure 2 summarizes the main relopathies according to their pathophysiology, and Table 2 provides an extended list of NF-κB activation syndromes.

**Table 2:** Main monogenic relopathies/NF-κB activation syndromes

Disorder	Gene	Inheritance	OMIM	Clinical features
TRAPS	*TNFRSF1A*	AD	142680	Fever, abdominal pain, migratory rash with fasciitis, periorbital rash, serositis
PGA/BS	*NOD2*	AD	186580	Granulomatous dermatitis, uveitis, synovitis, granulomatous arthritis, neuropathy
HA20	*TNFAIP3*	AD	616744	Arthralgia, mucosal ulcers, ocular inflammation, BD-like manifestations
ORAS	*OTULIN*	AR	615712	Early-onset recurrent fever, panniculitis, neutrophilia, diarrhea, arthritis
HOIL-1 deficiency	*HOIL-1 (RBCK1)*	AR	610924	Fever, recurrent infections, hepatosplenomegaly, amylopectin deposition, specific antibody defect
HOIP deficiency	*HOIP (RNF31)*	AR	612487	Fever, recurrent infections, hepatosplenomegaly, amylopectin deposition, specific antibody defect, lymphangiectasia
NLRP12-AID	*NLRP12*	AD GOF	611762	Cold urticaria, arthralgia and fever
RelA haploinsufficiency	*RELA*	AD	618287	Oral and gastrointestinal ulcers, cytopenias and lymphoproliferative disease, BD-like manifestations
NEMO-NDAS	*IKBKG (NEMO)*	XL	NA	Fever, rash, systemic inflammation, infections, panniculitis, uveitis, hepatosplenomegaly, ectodermal dysplasia, CNS involvement
ELF4 deficiency	*ELF4*	XL	301074	Early-onset IBD/mucosal autoinflammation, fever, ulcers, BD-like manifestations
CRIA	*RIPK1*	AD	618852	Fever, adenomegaly, hepatosplenomegaly, arthralgia, ulcers, gastrointestinal changes
ADAM17 deficiency	*ADAM17*	AR	614328	Early-onset colitis, rash, serious infections

AD (autosomal dominant); AR (autosomal recessive); BD (Behçet’s disease); CNS (central nervous system); CRIA (cleavage-resistant receptor-interacting protein kinase 1-induced autoinflammatory); GOF (gain-of-function); HA20 (haploinsufficiency of A20); IBD (inflammatory bowel disease); NA (not available); NEMO-NDAS (NF-κB essential modulator-deleted exon 5 autoinflammatory syndrome); NLRP12-AID (NACHT, LRR and PYD domains-containing protein 2-associated autoinflammatory disease); ORAS (OTU deubiquitinase with linear linkage specificity-related autoinflammatory syndrome); PGA/BS (pediatric granulomatous arthritis/Blau syndrome); TRAPS (tumor necrosis factor receptor-associated periodic syndrome); XL (X-linked)

### TNF receptor–associated periodic syndrome (TRAPS)

TRAPS originates from autosomal dominant mutations affecting the *TNFRSF1A* (TNF receptor superfamily member 1A) gene, which encodes TNF receptor 1. These mutations induce the dysregulation of critical cysteine‒cysteine disulfide bond formation within the receptors, leading to the accumulation of misfolded receptors in the endoplasmic reticulum. These aberrant proteins promote an increase in NF-κB activation and reactive oxygen species (ROS) generation and compromised autophagy [26].

Clinical manifestations of TRAPS typically emerge during childhood or adolescence. Flares are prolonged, lasting 2–4 weeks. Abdominal pain stemming from peritoneal inflammation is a prominent feature of TRAPS, often mirroring the surgical acute abdomen-like presentations observed in FMF. Myalgia is attributed to monocytic fasciitis, and affected patients can exhibit migratory tendencies, commonly accompanied by an erysipeloid rash. Additionally, ocular manifestations such as periorbital edema or conjunctivitis are frequently encountered. As TRAPS involves a TNF receptor defect, TNF inhibitors, mainly etanercept, becomes a therapeutical option. Noteworthy, etanercept is only partially effective, suggesting that the hypothesis of TNF pathway hyper activation may be possibly one of many mechanisms in TRAPS pathophysiology [27]. IL-1 blockade such as canakinumab, which is FDA-approved for the treatment of TRAPS, remains a cornerstone of treatment [28].

### Pediatric granulomatous arthritis/Blau syndrome (PGA/BS)

PGA/BS is an autosomal dominant monogenic disease characterized by GOF variants in *NOD2* (nucleotide-binding oligomerization domain-containing protein 2), which induce the hyperactivation of several intracellular proinflammatory molecular pathways, especially NF-κB. Moreover, uncontrolled production of IFNg leads to chronic inflammation and granuloma formation [22]. The prevalence of PGA/BS remains unknown, as many cases are undiagnosed or misclassified as other SAIDs. In its classic manifestation, patients usually develop a triad of arthritis, dermatitis, and uveitis. Typically, rash is the first symptom to appear, often in the first year of life and as an ichthyosiform erythematous rash. Between 2 and 4 years of age, polyarthritis is typically observed, with large synovial effusion more commonly affecting the wrists, although other joints, such as the knees, ankles, and proximal interphalangeal region, can also be affected. Uveitis, which manifests as insidious granulomatous iridocyclitis with potential progression to severe panuveitis, develops in 60–80% of patients at approximately 4 years of age. Currently, the most recommended pharmacological therapies for PGA/BS include corticosteroids and disease-modifying anti-rheumatic drugs, such as methotrexate. TNF inhibitors have also shown efficacy in controlling disease activity and preventing long-term complications.

### Haploinsufficiency of A20 (HA20)

*TNFAIP3* (tumor necrosis factor, α-induced protein 3) encodes A20, the main modulator of the canonical NF-κB pathway [29]. Zhou et al. [30] described six distinct germline mutations in *TNFAIP3* that cause pathergy, bipolar ulcers (oral and genital), uveitis, retinal vasculitis, and gastrointestinal involvement, thus becoming the first monogenic form of Behçet’s disease (BD) described [31]. Interestingly, some patients also exhibit clinical manifestations of systemic lupus erythematosus, such as thrombocytopenia, malar rash, polyarthritis, and circulating autoantibodies. These mutations display a pattern of haploinsufficiency inheritance and lead to uncontrolled NF-κB pathway activation and proinflammatory cytokine release. New cases have been recently reported and include various clinical manifestations as part of the HA20 phenotype: recurrent fever, atrophic gastritis, autoimmune thyroiditis, interstitial pneumopathy, nephropathy, and hepatopathy, among others.

Haploinsuficiency of A20 (HA20) patients usually experience an early onset of symptoms (mean at 14 years, with more than 70% before 10 years of age) and a marked increase in acute phase reactants. Additionally, fever, gastrointestinal involvement, and a positive family history (more than 80% of cases) are also common. Most patients respond to colchicine, making it a good option for first-line treatment. Corticosteroids are used in most cases, and anecdotal reports in refractory patients suggest the benefit of anti-IL1, anti-TNF, and mycophenolate mofetil, among other immunosuppressants [32].

### OTULIN-related autoinflammatory syndrome (ORAS)

OTULIN (OTU deubiquitinase with linear linkage specificity) is an important regulator of the NF-κB pathway. Loss-of-function (LOF) mutations and a recessive inheritance pattern of *OTULIN* have been associated with an early-onset autoinflammatory phenotype, featuring recurrent fever, arthritis, gastroenteritis, erythematous rash, subcutaneous nodules, and lipodystrophy. Similarly to HA20, ORAS is associated with aberrant NF-κB pathway hyperactivation. Reports of new cases in the future may expand the spectrum of ORAS manifestations [33].

### Linear ubiquitin chain assembly complex (LUBAC) component deficiency

LUBAC complex is composed of three proteins: HOIP (HOIL-1 interacting protein), HOIL-1 (heme-oxidized IRP2 ubiquitin ligase 1) and SHARPIN (SHANK-interacting protein like 1). LUBAC ubiquitinates protein targets of the canonical NF-κB pathway and therefore modulates inflammasome signaling and activation. HOIL-1 and HOIP deficiencies are recessive inherited diseases that result in the dysfunction of the entire complex. LUBAC deficiency impairs NF-κB regulation in fibroblasts and B cells, leading to primary antibody deficiency and recurrent bacterial infections. On the other hand, phagocytes from these patients respond to IL-1 stimulation via proinflammatory cytokine release and an autoinflammatory phenotype. Clinical manifestations include an eczematous rash, gastrointestinal manifestations, and oral ulcers. Notably, although muscular amylopectinosis has been observed in some patients, its etiology has not been fully elucidated [33].

### RelA haploinsufficiency (RelA-HI)

*RELA* encodes the NF-κB subunit RelA (p65). In 2017, a hypomorphic heterozygous mosaic variant in a canonical splicing site of *RELA* (c599 + 1G > A, p.Q162delE6fs12) resulting in RelA haploinsufficiency, was found in four cases of the same family with bipolar mucocutaneous ulcerations and early-onset colitis. Interestingly, all clinical manifestations were exclusively evidenced when the variant was expressed in fibroblasts [34]. In 2020, a new heterozygous mutation (p.H487Tfs7) leading to RelA-HI was associated with a BD phenotype (oral and genital ulcers and cutaneous pustulosis) with or without neuromyelitis optica [35]. In 2022, an additional frameshift variant, p.T349Lfs*13, was detected in a multigenerational family with a BD mucocutaneus phenotype [36]. Hence, similar to HA20, RelA-HI has also been considered a monogenic form of BD.

## Autoinflammatory disorders induced by IL-18/IL-36 signaling pathway defects

**Fig. 3:**
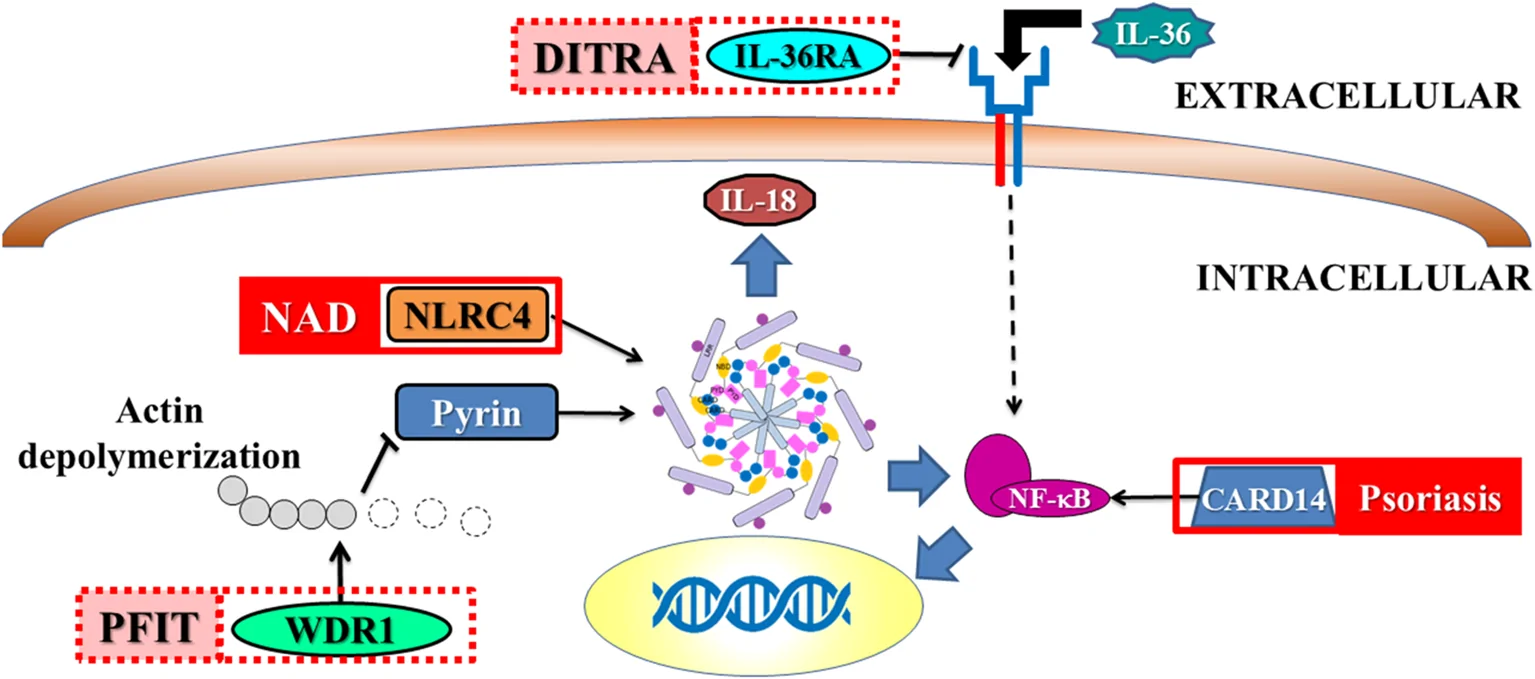
Main IL-18/IL-36 pathway defect-inducing autoinflammatory disorders classified according to pathophysiology. Pyrin function is modulated by cytoskeleton formation, especially the actin polymerization/depolymerization process, which is regulated by WDR1 function. This process mainly induces the IL-18-producing inflammasome, similar to the NLRC4 inflammasome. The IL-18/IL-36 pathway is critical for keratinocyte formation. Extracellular modulation of the IL-36 pathway is mainly modulated by IL-36RA, and intracellular signaling is mediated by CARD14-induced NF-κB. Red squares depict the main syndromes and their respective molecular targets classified as gain-of-function (continuous line) and loss-of-function (dashed line)-causing mutations. CAMPS (CARD14-mediated psoriasis); CARD14 (caspase recruitment domain family member 14); DITRA (deficiency of IL-36RA); GOF (gain-of-function); IL-36RA (IL-36 receptor antagonist); NAD (NLRP4 autoinflammatory disease); NLRP4 (NLR family CARD domain containing 4); PFIT (autoinflammatory periodic fever, immunodeficiency, and thrombocytopenia); WDR1 (WD repeat-containing protein 1)

Disorders resulting from defects in the IL-18 and IL-36 signaling pathways that disrupt the intricate balance of cytokine signaling encompass a spectrum of autoinflammatory conditions characterized by dysregulated immune responses, uncontrolled inflammation, and tissue damage, mainly in the skin. The cytokines IL-18 and IL-36 play crucial roles in orchestrating cutaneous innate and adaptive immune responses, modulating inflammatory cascades, and maintaining tissue homeostasis. Defects in these signaling pathways result in the hyperactivation of downstream effectors, such as NF-κB and MAPK (mitogen-activated protein kinase), culminating in increased proinflammatory cytokine production and immune cell infiltration. Figure 3 summarizes the main IL-18/IL-36 signaling pathway defect-induced autoinflammatory disorders according to pathophysiology, and Table 3 provides an extended list of these syndromes.

**Table 3:** Main monogenic IL-18/IL-36 signaling pathway defect-induced autoinflammatory disorders

Disorder	Gene	Inheritance	OMIM	Clinical features
DITRA	*IL-36RN*	AR	614204	Pustular psoriasis, fever
NAD	*NLRC4*	AD GOF	616050/616115	Enterocolitis, rash, fever and macrophage activation syndrome
PFIT	*WDR1*	AR	604734	Fever, infection, oral inflammation, perianal ulcers
CAMPS	*CARD14*	AD	602723	Familial psoriasis

AD (autosomal dominant); AR (autosomal recessive); CAMPS (caspase recruitment domain family member 14-mediated psoriasis); DITRA (deficiency of IL-36 receptor antagonist); GOF (gain-of-function); NAD (NLR family CARD domain containing 4 autoinflammatory disease); PFIT (autoinflammatory periodic fever, immunodeficiency, and thrombocytopenia)

### Deficiency of the IL-36 receptor antagonist (DITRA)

DITRA is a rare autosomal recessive autoinflammatory disorder caused by mutations in *IL36RN*, which encodes an extracellular IL-36 receptor antagonist. This dysregulation results in impaired inhibition of the IL-36 receptor and, consequently, of the whole pathway, triggering excessive production of proinflammatory cytokines. Clinically, DITRA patients present with recurrent episodes of skin inflammation characterized by erythematous plaques and sterile pustules, resembling generalized pustular psoriasis. Patients may also experience fever and musculoskeletal symptoms. Current therapeutic strategies include systemic medications such as acitretin, methotrexate, and cyclosporine, which target the underlying inflammatory process. Biological agents that inhibit the IL-17 and IL-1 pathways have also shown efficacy in treating DITRA. Additionally, ongoing research is exploring targeted therapies specifically directed at components of the IL-36 pathway, and the results may offer promising avenues for personalized treatment for individuals with DITRA [37].

### CARD14-mediated psoriasis (CAMPS)

CAMPS is an autosomal dominant disorder characterized by heterozygous mutations in *CARD14* (caspase recruitment domain family member 14), leading to dysregulated activation of the NF-κB signaling pathway. This dysregulation triggers an exaggerated cutaneous inflammatory response, resulting in the development of psoriasis-like skin lesions. CAMPS patients often present with erythematous plaques covered with silvery scales, often accompanied by itching and pain. Nail involvement, such as pitting and onycholysis, is also common. In some cases, CAMPS may be associated with psoriatic arthritis, which manifests as joint pain, swelling, and stiffness [38].

Topical therapies, including corticosteroids, vitamin D analogs, and topical calcineurin inhibitors, are typically used for mild to moderate cases. For more severe or refractory cases, systemic therapies such as methotrexate, cyclosporine, or oral retinoids may be prescribed. Biological agents targeting specific cytokines involved in the inflammatory cascade, such as TNFα, IL-17, or IL-23, have also shown efficacy in managing CAMPS [39].

### NLRC4 autoinflammatory disease (NAD)

NAD is an autosomal dominant disorder caused by GOF mutations in *NLRC4* (NLR family CARD domain containing 4), resulting in the dysregulation of the NLRC4 inflammasome pathway. This dysregulation culminates in uncontrolled activation of innate immune responses, triggering excessive production of IL-1β and, mainly, IL-18. NAD patients generally present with recurrent episodes of systemic inflammation, typically characterized by fever, cold-induced urticarial rash, joint pain, and gastrointestinal symptoms such as abdominal pain, diarrhea, and enterocolitis. Patients may also develop hepatosplenomegaly, lymphadenopathy and recurrent macrophage activation syndrome [40]. Corticosteroids, especially at high doses, are often used to manage acute severe flares. For severe cases, biological agents targeting IL-1β have shown efficacy in reducing inflammation and improving symptoms. Additionally, IL-18 binding protein may be considered a therapeutic option.

### Autoinflammatory periodic fever, immunodeficiency, and thrombocytopenia (PFIT)

PFIT is a rare autosomal recessive disorder characterized by recurrent episodes of fever, immunodeficiency, and a low platelet count. This condition arises from biallelic LOF mutations in *WDR1* (WD repeat domain 1), which disrupt the normal function of the WDR1 protein in actin filament dynamics and cellular processes [41]. The main consequence is abnormal activation of the pyrin inflammasome, leading to excessive production of IL-1β and IL-18.

PFIT patients present with episodic fevers lasting several days, accompanied by systemic symptoms such as fatigue, malaise, and arthralgia. Immunodeficiency often manifests as hypogammaglobulinemia and predisposes individuals to recurrent infections, while thrombocytopenia increases the risk of bleeding complications.

Immunosuppressive medications, such as corticosteroids, or biological immunomodulators, such as IL-1 blockers and TNFα inhibitors, may be prescribed to control autoinflammatory episodes. Due to its pathophysiology, IL-18 blockade may be a promising targeted-therapy for PFIT, although additional safety and efficacy studies are still needed to prove this hypothesis. Additionally, intravenous immunoglobulin replacement therapy may be used to boost immune function and prevent infections.

## Type I interferonopathies

Type I interferonopathies comprise a heterogeneous group of disorders sharing a common pathophysiology: uncontrolled production of type I IFNs, especially the α, β, and ε IFN isoforms. The term is derived from the observation of the protection induced by serum and cerebrospinal fluid from patients with Aicardi-Goutières syndrome (AGS) against infection of Madin-Darby bovine kidney cells by vesicular stomatitis virus, which was recently to be associated with IFNα activity [42, 43]. Therefore, AGS became the first type I interferonopathy identified. The subsequent demonstration of greater sensitivity for AGS diagnosis through the evaluation of interferon-stimulated gene (ISG) expression, a.k.a., the type I IFN signature, a typical feature observed among these patients, further supported this concept [44].

**Fig. 4:**
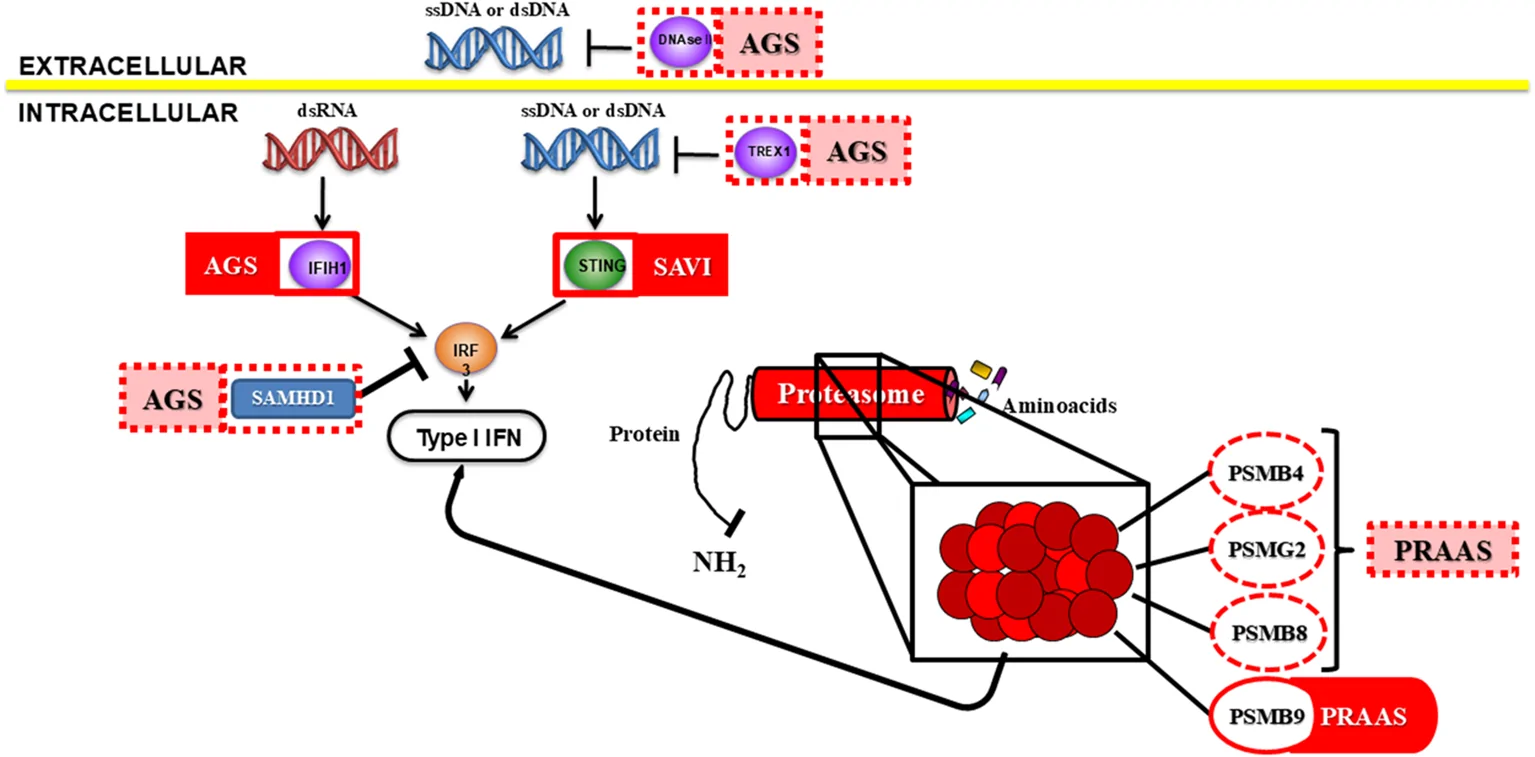
Main monogenic type I interferonopathies classified according to pathophysiology. Nucleic acids outside the cell nucleus are potent alarmins and may be cleaved and disabled by DNAse II (extracellular matrix) or TREX1 (intracellular). Moreover, type I IFN secretion is highly dependent on IFIH1- and STING-mediated IRF3 activation via double-stranded RNA and double- or single-stranded DNA, respectively. SAMHD1 is a pivotal modulator of IRF3. The proteasome is a tube-shaped protein complex of multiple heptamers composed of subunits (e.g., PSMB4, PSMG2, PSMB8 or PSMB9). The proteasome is involved in cleavage-induced protein activation and senescent peptide degradation. Type I IFN overproduction is also caused by mutations in different proteasome subunits, which culminate in intracellular oxidative stress. Red squares depict the main syndromes and their respective molecular targets classified as gain-of-function (continuous line) and loss-of-function (dashed line)-causing mutations. AGS (Aicardi-Goutières syndrome); dsDNA (double stranded desoxyribonucleic acid); dsRNA (double stranded ribonucleic acid); ssDNA (single stranded desoxyribonucleic acid); IFIH1 (IFN induced with helicase C domain 1); IFN (interferon); IRF3 (IFN regulatory factor 3); PRAAS (proteasome-associated autoinflammatory syndromes); PSMB4 (proteasome 20S subunit beta 4); PSMB8 (proteasome 20S subunit beta 8); PSMB9 (proteasome 20S subunit beta 9); PSMG2 (proteasome assembly chaperone 2); SAMHD1 (SAM and HD domain containing deoxynucleoside triphosphate triphosphohydrolase 1); SAVI (STING-associated vasculopathy of infancy onset); STING (stimulator of interferon genes); TREX1 (three prime repair exonuclease 1)

As type I IFN receptors mainly activate Janus kinase (Jak) 1/2 signaling, culminating in STAT1 (signal transducer and activator of transcription 1) phosphorylation, specific Jak 1/2 inhibitors such as ruxolitinib and baricitinib are mostly indicated as targeted therapies for all those disorders. Figure 4 summarizes the main type I interferonopathies according to pathophysiology, and Table 4 provides an extended list of these syndromes.

**Table 4:** Main monogenic type I interferonopathies

Disorder	Gene	Inheritance	OMIM	Clinical features
PRAAS	*PSMB4, PSMG2,* *PSMB8,* *PSMB9,* *PSMB10,* *POMP*	Digenic/AR/AD	602177/609702/256040/617591/619175/618048	Fever, joint contractures, lipodystrophy, cerebral calcifications, chilblain lupus, anemia
SPENCD	*ACP5*	AR	171640	Skeletal dysplasia, short stature, cytopenias, brain calcification, autoimmunity
SAVI	*STING (TMEM173)*	AD/AR GOF	612374/615934	Chilblain lupus, small vessel vasculitis, severe interstitial lung disease, arthritis
Aicardi-Goutières syndrome	*TREX-1, ADAR1,* *DNASE2,* *RNASEH2A, RNASEH2B, RNASEH2C, SAMHD1, IFIH1,* *LSM11,* *RNU7-1, USP18,* *STAT2*	AR/AD	606609/146920/126350/606034/610326/610330/606754/615846/619486/619487/607057/616636	Progressive encephalopathy, fever, cerebral calcifications, chilblain lupus, skin ulcers, hydrocephalus, seizures, autoimmunity
SMS	*IFIH1, DDX58a*	AD	615846/616298	Aortic and valve calcifications, dental abnormalities, osteopenia, idiopathic osteolysis of the extremities
COPA syndrome	*COPA*	AD	616414	Arthritis, interstitial lung disease, autoimmunity
DNASEIL13 deficiency	*DNASEIL13*	AR	614420	Very early-onset systemic lupus erythematosus, lupus nephritis, autoimmunity, urticaria, hypocomplementemic vasculitis
X-linked reticulate pigmentary disorder	*POLA1*	XL	301220	Reticular hyperpigmentation, gastrointestinal and pulmonary inflammatory disease, corneal ulcer, typical facial features
DEX	*ELF4*	XL	301074	Early-onset colitis, fever, ulcers, BD-like manifestations
OAS1 deficiency	*OAS1*	AD GOF	164350	Pulmonary alveolar proteinosis, rash
CDC42 deficiency	*CDC42*	AD	616737	Pancytopenia, rash, hepatosplenomegaly, bone marrow fibrosis/proliferation, HLH, enterocolitis, developmental delay
ATAD3A deficiency	*ATAD3A*	AD/AR	617183	Developmental delay, spasticity, elevated type 1 ISG

AD (autosomal dominant); AR (autosomal recessive); BD (Behçet’s disease); COPA (COPI coat complex subunit alpha); DEX (deficiency of ELF-4, X-linked); GOF (gain-of-function); HLH (hemophagocytic lymphohistiocytosis); IFN (interferon); ISG (interferon stimulated genes); PRAAS (proteasome-associated autoinflammatory syndromes); SAVI (STING-associated vasculopathy of infancy onset); SPENCD (spondyloenchoncdrodysplasia); SMS (Singleton-Merten syndrome); XL (X-linked)

### Aicardi-Goutières syndrome (AGS)

AGS is the prototype type I interferonopathy and, although monogenic, AGS can stem from defects in different genes. *TREX1* (three prime repair exonuclease 1) is a key gene in AGS, accounting for 25% of cases, although variants in other genes, such as *RNASEH2A-C, SAMHD1* (SAM and HD domain containing deoxynucleoside triphosphate triphosphohydrolase 1), *ADAR*, among others, have also been associated with AGS (Table 4). Although spectral, AGS typically emerges within the first 6 months of life and is characterized by progressive encephalopathy with basal ganglia calcification, white matter alterations, neurodevelopmental delay, hepatosplenomegaly, arthritis, cutaneous manifestations (usually chilblain lupus), oral ulcers, thrombocytopenia, and leukopenia. Anti-double-stranded DNA (dsDNA) antibodies or antibodies to extractable nuclear antigens (ENA) may be present [45]. In addition to Jak 1/2 inhibitors, recently approved [46], immunobiologicals against type I IFN receptors may be therapeutic options [47].

### STING-associated vasculopathy with onset in infancy (SAVI)

SAVI is an autosomal dominant interferonopathy caused by GOF variants in stimulator of interferon genes (STING). Clinically, patients with SAVI present with peripheral vasculitis, with diverse lesion types in cold exposed areas, and ischemia of the auricular pavilion, nose, hands, and feet. Patients with SAVI can also present with severe inflammatory lung disease, a feature not observed in other type I interferonopathies [48]. Peripheral skin and lung disease are usually exclusive phenotypes. Along with SAVI syndrome, COPI coat complex subunit alpha (COPA) syndrome, whose pathophysiology has recently been associated with constitutive activation of STING signaling, is the only type I interferonopathy that predominantly involves the lungs. SAVI might also resemble juvenile systemic lupus erythematosus in patients that feature polyarthritis or those with positive autoantibodies, as the SAVI facial rash can be confused with malar rash. Acute-phase reactants may rise during disease activity episodes, akin to other SAIDs. The presence of autoimmunity with autoantibodies may lead to a misdiagnosis of lupus, yet a type I IFN signature greatly aids in identifying this entity. An interferon-blocking agent, such as JAK-STAT pathway inhibitors are the drugs of choice and should be initiated promptly due to the high mortality of this disease [49]. However, SAVI is generally unresponsive to conventional immunosuppressants and only partially responsive to JAK1/2 inhibitors. The use of type I IFN receptor blockers, anifrolumab, and antifibrotic drugs, such as nintedanib and pirfenidone, may be promising, especially if approval for young children is obtained. It is still unclear if lung transplantation in end-stage respiratory failure would be beneficial in SAVI patients. Finally, direct inhibition of STING might be a promising therapeutic option in the future [50, 51].

### Proteasome-associated autoinflammatory syndromes (PRAAS)

PRAAS are a subgroup of type I interferonopathies resulting from mutations that affect proteasome components, leading to ubiquitination impairment, dysregulated protein degradation and the accumulation of truncated proteins. This disruption triggers intracellular oxidative stress and uncontrolled stimulation of the JAK-STAT pathway, culminating in type I IFN overproduction.

PRAAS can also be referred to as CANDLE (chronic atypical neutrophilic dermatosis with lipodystrophy and elevated temperature) syndrome represents the prototypical clinical phenotype of this subgroup [52]; however, a similar clinical phenotype under the eponym “Nakajo-Nishimura syndrome” was simultaneously described by another group, who reported autosomal dominant inheritance induced by heterozygous variants in *PSMB8* (proteasome subunit beta type-8) [53]. All these conditions revealed a single disorder and were grouped as PRAAS. Subsequent reports additionally linked the syndrome to biallelic variants (autosomal recessive) and even digenic inheritance, in which two mutations in two different genes (usually *PSMB4, PSMB9, PSMB10, PSMG2* [proteasome assembly chaperone 2], and *POMP* [proteasome maturation protein] synergistically determine the clinical phenotype. Other genes associated with PRAAS are *PSMA5* and the gene encoding proteasome assembly proteins *POMP* (AD inheritance). More recently, monoallelic variants in *PSMB9* or *PSMB10* as single genes have been associated with an immune dysregulatory phenotype. Patients with CANDLE can present with intermittent febrile episodes, chilblain lupus (typically acral), periorbital edema, neutrophilic dermatosis, lipodystrophy, and central nervous system manifestations with basal ganglia calcifications. JAK-STAT pathway inhibitors and type I IFN receptor blockers appear to be optimal therapeutic strategies [52].

## Anti-inflammatory signaling pathway impairment-induced autoinflammatory disorders

**Fig. 5:**
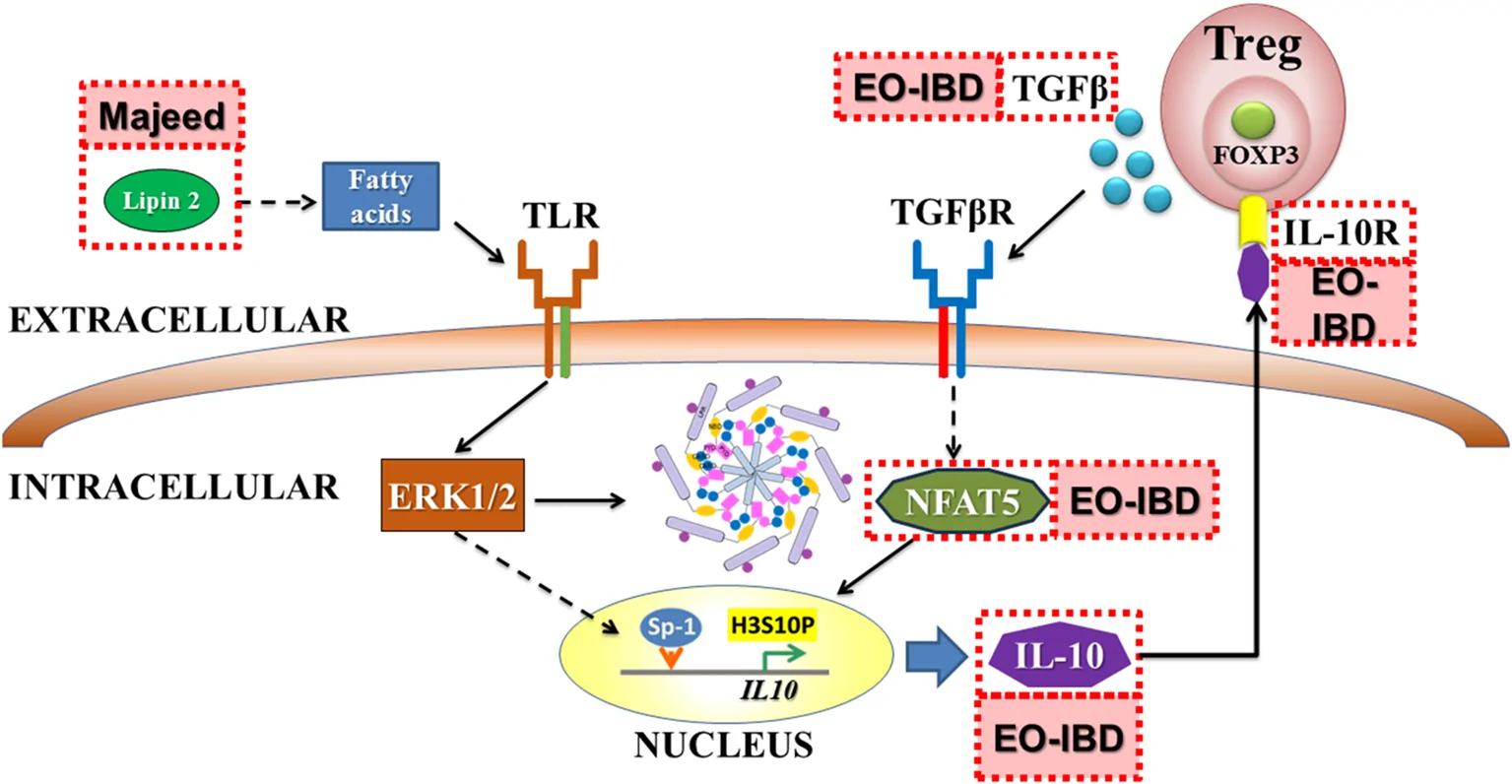
Main monogenic anti-inflammatory signaling pathway impairment-induced autoinflammatory disorders classified according to pathophysiology. Lipin 2 is a regulator of fatty acid production, which, in turn, may also be an alarmin at inflammatory sites, as it is recognized by TLRs. ERK1/2 not only mediates the inflammatory response but also induces the secretion of IL-10, which is a potent anti-inflammatory Treg-stimulating cytokine. Treg responses include the secretion of TGFβ, another pivotal anti-inflammatory cytokine, which signals intracellularly through NFAT5 activation. Red squares depict the main syndromes and their respective molecular targets classified as loss-of-function (dashed line)-causing mutations. EO-IBD (early-onset inflammatory bowel disease); ERK1/2 (extracellular signal-regulated kinase 1/2); FOXP3 (forkhead box P3); H3S10P (histone H3 at Ser10); IL-10R (interleukin-10 receptor); NFAT5 (nuclear factor of activated T cells 5); Sp-1 (specificity protein 1); TGFβ (transforming growth factor beta); TLR (Toll-like receptor); Treg (regulatory T cell)

Monogenic anti-inflammatory signaling pathway impairment-induced autoinflammatory disorders encompass a spectrum of conditions characterized by disruption of the IL-10 and transforming growth factor β (TGFβ) anti-inflammatory pathways, which are critical modulatory cytokines that play a key role in regulating immune homeostasis and suppressing excessive inflammation. The dysfunction of these pathways can result from genetic mutations affecting cytokine secretion, membrane receptor function or downstream signaling molecules. Figure 5 summarizes the main monogenic anti-inflammatory signaling pathway impairment-induced autoinflammatory disorders according to pathophysiology, and Table 5 provides an extended list of these syndromes.

**Table 5:** Main monogenic anti-inflammatory signaling pathway impairment-induced autoinflammatory disorders

Disorder	Gene	Inheritance	OMIM	Clinical features
Majeed syndrome	*LPIN2*	AR	609628	Aseptic osteomyelitis, fever, rash, dyserythropoietic anemia
IL-10 deficiency	*IL-10*	AR	124092	Early-onset IBD
IL-10 R deficiency	*IL-10RA,* *IL10-RB*	AR	146933/123889	Early-onset IBD
NFAT5-HI	*NFAT5*	AD	604708	Early-onset IBD, recurrent sinopulmonary infections
TGFB1 deficiency	*TGFB1*	AR	618213	Early-onset IBD, immunodeficiency, recurrent viral infections, microcephaly, and encephalopathy

AD (autosomal dominant); AR (autosomal recessive); IBD (inflammatory bowel disease); IL-10R (IL-10 receptor); NFAT5-HI (NFAT5 haploinsufficiency)

Primary causes of IL-10 signaling defects include genetic LOF mutations affecting IL-10 itself [54] or IL-10 receptor subunits (IL-10RA and IL-10RB) [55, 56]. Monogenic causes of TGFβ signaling defects involve LOF variants in the genes encoding cytokines [57] and in the main transcription factor NFAT5 (nuclear factor of activated T cells 5) [58, 59]. All these disorders are autosomal recessive, except for NFAT5 haploinsufficiency, which has an autosomal dominant inheritance pattern. The absence or impairment of this important anti-inflammatory signaling axis disrupts the balance between proinflammatory and anti-inflammatory signals, resulting in sustained activation of innate and adaptive immune responses. Ultimately, this dysregulation culminates in regulatory T cell dysfunction, chronic inflammation, tissue damage, and systemic manifestations.

Clinical presentations vary depending on the subtype and severity of the disease. Interestingly, however, all disorders grouped in this classification share a clinical feature—early-onset inflammatory bowel disease (EO-IBD), which typically presents before 6 years of age. EO-IBD commonly manifests as abdominal pain, diarrhea, rectal bleeding, and weight loss. Extraintestinal manifestations such as arthritis, rashes, and delayed growth may also occur. More severe presentations start even earlier, in children younger than 2 years old, and are recognized as “very EO-IBD” (VEO-IBD). VEO-IBD often manifests as more severe symptoms, including bloody diarrhea, multiple enterocutaneous fistula formations, failure to thrive, treatment-resistant colitis, and systemic inflammation. Patients may exhibit developmental delay, anemia, and malnutrition.

The clinical manifestations of IL-10 signaling defects vary widely but commonly include recurrent fever, rash, arthritis, gastrointestinal symptoms (such as diarrhea and abdominal pain), hepatosplenomegaly, lymphadenopathy, immunodeficiency with susceptibility to infections, and autoimmune features. NFAT5 haploinsufficiency also typically manifests as immune dysregulation, including recurrent infections, and autoimmune manifestations (e.g., autoimmune hemolytic anemia). Patients may also exhibit renal abnormalities such as nephrogenic diabetes insipidus and electrolyte disturbances due to impaired osmoregulation. TGFB1 deficiency, on the other hand, primarily manifests as immunodeficiency with recurrent infections, particularly affecting the respiratory and gastrointestinal systems. Additionally, patients with TGFB1 deficiency may develop autoimmune hepatitis.

Therapeutic approaches for these conditions aim to induce and maintain remission, alleviate symptoms, and improve quality of life. Treatment options include amino salicylates, corticosteroids, immunosuppressants (e.g., azathioprine and methotrexate), and biological agents targeting TNFα or integrins. Additionally, targeted therapies that modulate the IL-10 signaling pathway, such as IL-10 receptor agonists or JAK inhibitors, hold promise for restoring immune homeostasis. Nutritional therapy and exclusive enteral nutrition may also be utilized, particularly for pediatric patients. Personalized treatment plans tailored to the patient’s disease phenotype, severity, and response to therapy are essential for optimizing outcomes. Early intervention and close monitoring are crucial to prevent disease progression and complications. Notably, due to the severe systemic inflammation caused by monogenic anti-inflammatory signaling pathway impairment-induced autoinflammatory disorders, a significant portion of these patients may require hematopoietic stem-cell transplantation.

Finally, a rare autosomal recessive SAIDs may also be classified as an anti-inflammatory signaling pathway impairment-induced autoinflammatory disorder—Majeed syndrome, a condition characterized by the triad of chronic recurrent multifocal osteomyelitis, congenital dyserythropoietic anemia, and neutrophilic dermatosis [60]. The pathophysiology of Majeed syndrome involves LOF mutations in *LPIN2* (lipin-2), leading to the dysregulation of fatty acid metabolism. As some substrates derived from fatty acid metabolic pathways act as alarmins, the accumulation of these compounds hyperactivates the innate immune system. Clinically, patients present with recurrent episodes of bone pain and swelling due to osteomyelitis, often beginning in infancy or early childhood, and dyserythropoietic anemia. Neutrophilic dermatosis typically manifests as skin lesions such as lipodystrophy, pustules, nodules, or abscesses. Treatment strategies for Majeed syndrome are similar to those for chronic recurrent multifocal osteomyelitis: nonsteroidal anti-inflammatory drugs may help control the pain and inflammation associated with osteomyelitis but fail in more than 50% of patients after 2–3 years; corticosteroids are sometimes used for acute flares of inflammation; and methotrexate, bisphosphonates (mainly pamidronate), TNF inhibitors or IL-1 blockers are used in severe cases.

## Miscellaneous and polygenic SAIDs

As this area has evolved, novel genetic defects with intricate functions within multiple inflammatory pathways are being described. The miscellaneous conditions presented here have a clear autoinflammatory phenotype characterized by dysregulated innate immune responses but may involve multiple genetic factors, a complex inheritance pattern or a unique pathophysiology. Therefore, herein, we grouped all these disorders as miscellaneous and polygenic SAIDs, probably representing the largest group of diseases according to our classification (Table 6).

**Table 6:** Miscellaneous and polygenic autoinflammatory diseases

Disorder	Gene	Inheritance	OMIM	Clinical features
ADA2 deficiency	*ADA2*	AR	607575	Polyarteritis nodosa, early-onset ischemic strokes, fever and cytopenias
VEXAS syndrome	*UBA1*	XL (somatic)	301054	Late-onset treatment refractory inflammatory syndrome (fevers, cytopenias, dysplastic bone marrow, interstitial nephritis, chondritis, vasculitis)
Schnitzler syndrome	*NA*	NA	NA	Chronic urticarial rash, fever, bone pain, monoclonal gammopathy
PFAPA syndrome	*NA*	NA	NA	Recurrent fever, aphthous stomatitis, pharyngitis, tonsilitis, cervical adenitis, abdominal pain
CNO/CRMO	*Multiple*	Multiple	612852/259680	Recurrent painful aseptic bone lesions
SAPHO syndrome	*NA*	NA	NA	Synovitis, acneiform lesions, neutrophilic dermatosis, hyperostosis, osteitis
Still’s disease	*NA*	NA	NA	Recurrent fever, polyarthritis, salmon-colored rash associated with body temperature spikes, elevated inflammatory markers
TRIAD	*NA*	NA	NA	Fever, erythema nodosum, gastrointestinal manifestation, myelodysplastic syndrome
LAVLI	*LYN*	AD GOF	620376	Cutaneous small vessel vasculitis, periorbital edema, hepatosplenomegaly, periostitis, thrombocytopenia
NCKAP1L deficiency	*NCKAP1L*	AR LOF	618982	Rash, skin abscesses, atopy, ulcers, recurrent infections, fever, severe malnutrition, lymphoproliferation
XLP syndrome	*XIAP*	LX	300079	Splenomegaly, lymphoproliferation, colitis, hepatitis, hypogammaglobulinemia, HLH
SIFD syndrome	*TRTN1*	AR	616084	Fever, developmental delay, seizures, microcytic anemia and hypogammaglobulinemia
RIPK1 deficiency	*RIPK1*	AD/AR	618852/618108	Colitis, recurrent infections, progressive polyarthritis, fever, lymphadenopathy, hepatosplenomegaly, ulcers
TGFB1 deficiency	*TGFB1*	AR	618213	Recurrent viral infections, microcephaly, encephalopathy, early-onset colitis
NFAT5 haploinsufficiency	*NFAT5*	AD	604708	Severe early-onset colitis, hypogammaglobulinemia, elevated IgE
TBK1 deficiency	*TBK1*	AR	NA	Polyarthritis, vasculitis, developmental delay

AD (autosomal dominant); AR (autosomal recessive); CNO/CRMO (chronic nonbacterial osteomyelitis/chronic recurrent multifocal osteomyelitis); CNS (central nervous system); HLH (hemophagocytic lymphohistiocytosis); JIA (juvenile idiopathic arthritis); LAVLI (Lyn kinase-associated vasculopathy and liver fibrosis); NA (not available); SAPHO (synovitis, acne, pustulosis, hyperostosis, and osteitis); SIFD (sideroblastic anemia with B-cell immunodeficiency, periodic fevers, and developmental delay); TRIAD (trisomy 8-associated autoinflammatory disease); VEXAS (vacuoles, E1 enzyme, X-linked, autoinflammatory, somatic); XLP (X-linked lymphoproliferative disease)

### Adenosine deaminase 2 (ADA2) deficiency

ADA2 deficiency is a monogenic disease caused by biallelic (autosomal recessive) LOF mutations in *ADA2*. Although the disorder has been described as an autoinflammatory vasculitis, the clinical phenotype is heterogeneous and depends on ADA2 activity levels, potentially resulting in cutaneous ulcerations, recurrent ischemic stroke, humoral immunodeficiency, fever, myalgia, livedo reticularis, variable degrees of bone marrow failure, and severe medium-vessel vasculitis, thus representing a monogenic form of polyarteritis nodosa. The homozygous mutation c.973–2A > G in *ADA2* has also been associated with an infantile-onset BD-like phenotype, manifesting as oral and genital ulcers, arthralgia, folliculitis, and erythema nodosum, along with an adequate therapeutic response to anti-TNF administration [61]. DADA2 pathogenesis remains unclear; nevertheless, one widely accepted theory suggests that ADA2 deficiency could skew the balance of macrophage subpopulations toward the inflammatory M1 subtype, leading to endothelial activation and vaso-occlusion. While anti-TNF therapy is the immunosuppressive treatment of choice [62], to the best of our knowledge no preference upon any specific drug is required, as different authors have been using TNF inhibitors interchangeably [63]. Indeed, a recent systematic review included 242 ADA2 deficient patients, of which 44% were under different anti-TNF therapy: etanercept (59.5%), adalimumab (22.1%) and infliximab (12.2%) [64]. As expected, TNF inhibitors were effective in 78.6% of patients. Nevertheless, refractory cases represent a significant proportion of patients and may require bone marrow transplantation [65].

### Chromosome 8 trisomy

Some clinical autoinflammatory phenotypes resembling BD have been occasionally associated with chromosome 8 trisomy, both in its constitutional mosaic form and in its acquired form, secondary to myelodysplastic syndrome. Patients with BD associated with trisomy 8 often present with fever and gastrointestinal involvement, with reports of erythema nodosum as well. Since the array of autoinflammatory clinical phenotypes associated with trisomy of chromosome 8 is not restricted to BD, these cases have recently been termed TRIAD (trisomy 8-associated autoinflammatory disease) [66]. The pathogenesis of this association is still unknown; however, based on the presence of trisomy, it is hypothesized that the increased copy number of one or more genes may be responsible.

### Periodic fever, aphthous stomatitis, pharyngitis, and cervical adenitis (PFAPA) syndrome

PFAPA syndrome is the most prevalent periodic fever syndrome in childhood [67]. Despite its prevalence, the genetic underpinnings of PFAPA are complex. Possible variants in the *IL12A, IL10, STAT4, CCR1-CCR3* and HLA risk alleles may play contributing roles. The correlation observed between aphthous stomatitis or ulcers and PFAPA syndrome hints at potential associated mutations, as recent studies indicate shared genetic risk variants among PFAPA syndrome, BD, and recurrent aphthous ulcers [68].

In a recent cohort of Israeli patients [69], PFAPA syndrome had a disproportionately greater representation in children of Mediterranean descent. Patients with PFAPA syndrome present with recurrent febrile episodes exhibiting strikingly regular timing, often accompanied by aphthous ulcers, pharyngitis, and/or cervical lymphadenitis during flare-ups [70, 71]. Although a predominant onset during childhood, few cases of PFAPA have already been described in adults. Diagnosis relies on clinical features and may be aided by updated classification criteria [72, 73]. Treatment encompasses symptomatic medication, continuous use of colchicine as a prophylactic medication, and the administration of corticosteroids during flare-ups. In addition, a possible association between recurrent use of corticosteroids during flare-ups and shorter intercrisis periods is observed in clinical practice. Rarely, TNF inhibitors or IL-1 blockers may be indicated in refractory patients. Tonsillectomy is occasionally indicated and may be effective in children with PFAPA syndrome [74].

### VEXAS syndrome

VEXAS syndrome, a recently described autoinflammatory disorder, poses a diagnostic challenge due to its heterogeneous clinical presentation and unique genetic underpinnings. The acronym VEXAS encapsulates its essential features: vacuoles, E1 enzyme, X-linked, autoinflammatory, and somatic. The characteristic vacuolation of myeloid and erythroid precursors in bone marrow specimens is a common finding [75].

The identification of somatic mutations in *UBA1*, which encodes ubiquitin-activating E1 enzyme, provides the genetic basis for VEXAS syndrome. This X-linked mutation predominantly affects hematopoietic cells, resulting in a mosaic pattern of disease presentation, which is more prevalent in males. *UBA1* variants lead to reduced ubiquitination and inflammatory pathway activation, resulting in systemic autoinflammatory manifestations [76].

The *UBA1* variant in VEXAS syndrome is a somatic mutation, meaning that it is not homogenously present in all tissue cells. Therefore, variants with allele frequencies below a threshold may be overlooked, revealing the necessity of a second-generation sensitive sequencing methodology of peripheral leucocytes or, preferably, of different tissues (including bone marrow) to improve diagnosis [77]. *UBA1* mutations may be mistaken for germline mutations, which are associated with X-linked spinal muscular atrophy 2 (SMAX2), an important diagnostic bias [78, 79].

The pathogenesis of VEXAS syndrome involves the dysregulation of ubiquitin pathways due to *UBA1* mutations. To our knowledge, most of the pathogenic mutations described involve substitutions of methionine at codon 41 (p.Met41), which disrupt the initiation of the transcription of cytoplasmic isoform UBA1b, halting its expression and creating an alternative initiation codon at p.Met67, leading to UBA1c with reduced catalytic activity [80, 81]. It has been hypothesized that this disruption results in the accumulation of misfolded proteins, endoplasmic reticulum stress and likely the activation of the unfolded protein response, culminating in neutrophil extracellular trap formation [75, 82, 83] and ultimately inducing aberrant immune responses and hematopoietic cell dysfunction.

Patients with VEXAS syndrome commonly present with a triad of hematological abnormalities, inflammatory rheumatic manifestations, and dermatological findings in men beyond the sixth decade of life. Hematological features mainly include myelodysplastic syndrome, but plasma cell dyscrasia, recurrent neutropenia, monocytopenia and other hematological malignancies have also been reported [84]. Systemic inflammation manifests as relapsing polychondritis, polyarteritis nodosa, fever, weight loss, arthralgia/arthritis, uveitis, scleritis, episcleritis, orbital masses, lung involvement, and lymphadenopathy [85]. Dermatological manifestations range from neutrophilic dermatoses to cutaneous vasculitis, often resembling those of other rheumatological conditions. Erythema nodosum, urticaria, erythematosus papules, periorbital edema and injection-site reactions have also been described.

VEXAS syndrome remains a rare condition, making precise estimates of its prevalence and incidence challenging. However, recent evidence indicates that *UBA1* pathogenic variants are present in 1 in 13,591 individuals and broadens the clinical phenotype of VEXAS syndrome [86]. The coexistence of inflammatory and hematological dysfunction presents a unique challenge in the management of VEXAS syndrome, which often requires a multidisciplinary approach. Management of the inflammatory component may require high-dose steroids, JAK inhibitors, and IL-1 and IL-6 blockers. For the subset of patients who become increasingly cytopenic and transfusion dependent, allogenic hemopoietic stem cell transplantation represents the only viable treatment option [83].

### Chronic recurrent multifocal osteomyelitis/chronic nonbacterial osteomyelitis (CRMO/CNO)

CRMO/CNO is a rare idiopathic bone SAIDs characterized by recurrent episodes of painful and aseptic bone lesions, mainly affecting the metaphysis of the long bones, jaw, spine, pelvis, and clavicle, with symmetric distribution in approximately 50% of patients. Most patients lack a monogenic etiology, albeit few well-described cases caused by heterozygous IL-1 receptor GOF variants, which induce IL-1 pathway hyperactivation, were recently reported [87]. Approximately 10% of patients develop the disease after the age of 20, with a higher prevalence in females. CRMO/CNO has an insidious course, with periods of exacerbation and remission that can persist for years and may be associated with arthritis or osteitis. Extraosseous manifestations are also described, usually involving the gastrointestinal tract, such as inflammatory bowel disease, and the skin, including palmoplantar pustulosis, acne, pyoderma gangrenosum, and psoriasis. Diagnosis is based on clinical, radiological, and histological criteria, with mandatory exclusion of infectious and neoplastic etiologies [87].

Synovitis, acne, pustulosis, hyperostosis, and osteitis (SAPHO) syndrome is a clinical entity within the spectrum of CRMO/CNO, commonly occurring between the third and fifth decades of life, with a preference for females. Although its exact incidence is unknown, it is estimated to affect 1 in 10,000 individuals in Europe [88]. Osteoarticular involvement is usually insidious, with the sternum, clavicle, and sternoclavicular joints being the most common involved sites (90% of cases). Additionally, the condition may or may not be associated with synovitis, sacroiliitis, or axial involvement. Cutaneous manifestations belong to the group of neutrophilic dermatoses, with palmoplantar pustulosis being the most common, although acne, psoriasis, and hidradenitis suppurativa are also described. Diagnosis is clinical and requires the exclusion of infectious and neoplastic conditions, as there are no specific biomarkers or well-established diagnostic criteria, despite reports of associations with HLA-B27 [89].

Nonsteroidal anti-inflammatory drugs and corticosteroids are generally indicated for patients in the acute phase of CRMO/CNO or SAPHO syndrome. In refractory cases, bisphosphonates and immunosuppressants such as methotrexate, sulfasalazine, and TNF inhibitors may be used. Additionally, anti-IL-17 is also a therapeutic option for SAPHO [90].

### Schnitzler syndrome

Schnitzler syndrome is a rare SAIDs initially described in 1972 [91]. Typically, it begins around the age of 55 and is characterized by chronic urticarial rash (100%), recurrent fever (72%), bone pain, arthralgia/arthritis usually in the hip (40%), and elevated inflammatory markers such as CRP and ESR [92]. Erythematous macules, papules, or discrete raised plaques, mostly located on the trunk and hips, often temporary and dynamic, appear mainly at night and persist for up to 48 hours. The main laboratory finding is monoclonal gammopathy of undetermined significance of the IgM type or, less commonly, IgG. Bone marrow evaluation findings are normal in 80% of patients, but lymphoproliferative disorder complications may occur over time [93]. The exclusion of other SAIDs, lymphoma and Waldenström macroglobulinemia is essential for the appropriate diagnosis of Schnitzler syndrome. First-line treatment involves IL-1 blockers and colchicine [92].

### Still’s disease

Still’s disease is an idiopathic SAIDs that usually affects young adults with similar clinical findings to systemic juvenile idiopathic arthritis. Typically, it is characterized by the clinical triad of daily spiking body temperature (>39 °C, <4-h duration, usually in the morning and evening), polyarthritis of large joints (wrist, knee, and hip), and a salmon-colored evanescent rash located on the trunk and proximal limbs, often preceded by constitutional symptoms. Lymphadenopathy, hepatosplenomegaly, and leukocytosis are frequently present [94].

The prevalence of Still’s disease is estimated to be 1–34 cases per 1,000,000, with no sex predilection. Although the incidence has a bimodal distribution, with the first peak occurring between 15 and 25 years of age and the second occurring between 36 and 46 years, the disease can occur at any age. Despite having similar initial features, patients with Still’s disease can have heterogeneous disease progression, as monophasic, polycyclic and chronic articular courses are recognized. Approximately one-third of patients develop chronic articular disease, with persistent synovitis, bone erosions, and a propensity for carpal ankylosis [95]. Macrophage activation syndrome is a life-threatening complication that occurs in up to 15% of patients. Macrophage activation syndrome can occur at any time during Still’s disease and may even be present at the time of disease onset. Patients with Still’s disease complicated with macrophage activation syndrome have continuous fever, cytopenias, liver dysfunction, and a tendency toward a low ESR due to progressive hypofibrinogenemia.

Elevated ESR, CRP, SAA and, typically, serum ferritin (>1000 ng/mL) are reported. The diagnosis of Still’s disease is primarily based on clinical findings and requires the exclusion of infectious, neoplastic, and other autoimmune disease or SAIDs. Moreover, Still’s disease should be included in the numerous differential diagnoses of fever of unknown origin.

Corticosteroids are the first-line treatment. Corticosteroid-sparing agents, particularly methotrexate, are often used and should be considered as early as possible. Treatment with IL-1 or IL-6 blockers tends to be associated with rapid control of disease activity. TNF inhibitors also have a good response [96].

## Conclusions

Although relatively uncommon, SAIDs merit significant consideration as crucial differential diagnoses within the realm of rheumatological diseases, particularly in pediatric and adolescent patients. Expedient genetic investigation is a pivotal diagnostic tool. However, with the increasing diagnostic and therapeutic possibilities and the consequent rise in survival rates among children and adolescents with autoinflammatory diseases, they often transition into adulthood while still afflicted by illness. Moreover, as highlighted above, certain autoinflammatory diseases may onset during adulthood, either induced by somatic mutations or through late manifestations of germline genetic variants in genes of low penetrance or with paucisymptomatic presentations, yet exacerbated during adulthood. In summary, advancing the understanding of genetic risk factors holds key significance in elucidating SAIDs pathophysiology, given the multiple identified genetic mutations and complex and intricate influences on multiple inflammatory pathways identified thus far. Further research in this domain is imperative for a comprehensive understanding.

## Data Availability

Data sharing is not applicable to this article, as no datasets were generated or analyzed during the current study.
